# Radiolabeled Cetuximab Conjugates for EGFR Targeted Cancer Diagnostics and Therapy ^†^

**DOI:** 10.3390/ph7030311

**Published:** 2014-03-05

**Authors:** Wiebke Sihver, Jens Pietzsch, Mechthild Krause, Michael Baumann, Jörg Steinbach, Hans-Jürgen Pietzsch

**Affiliations:** 1 Helmholtz-Zentrum Dresden-Rossendorf (HZDR), Institute of Radiopharmaceutical Cancer Research, Bautzner Landstraße 400, Dresden 01328, Germany; E-Mails: j.pietzsch@hzdr.de (J.P.); j.steinbach@hzdr.de (J.S.); h.j.pietzsch@hzdr.de (H.-J.P); 2 Department of Chemistry and Food Chemistry, Technische Universität Dresden, Dresden 01062, Germany; 3 Department of Radiation Oncology and OncoRay, Medical Faculty and University Hospital Carl Gustav Carus, Technische Universität Dresden, Dresden 01307, Germany; E-Mails: mechthild.krause@uniklinikum-dresden.de (M.K.); michael.baumann@uniklinikum-dresden.de (M.B.); 4 OncoRay—National Center for Radiation Research in Oncology, Medical Faculty and University Hospital Carl Gustav Carus, Technische Universität Dresden, Dresden 01307, Germany; 5 German Cancer Consortium (DKTK) Dresden and German Cancer Research Center (DKFZ), Heidelberg 69120, Germany; 6 HZDR, Institute of Radiation Oncology, Bautzner Landstraße 400, Dresden 01328, Germany

**Keywords:** EGFR, radiolabeled cetuximab conjugates, radioimmunotherapy (RIT), cancer theranostics, external beam radiotherapy (EBRT), endoradionuclide therapy

## Abstract

The epidermal growth factor receptor (EGFR) has evolved over years into a main molecular target for the treatment of different cancer entities. In this regard, the anti-EGFR antibody cetuximab has been approved alone or in combination with: (a) chemotherapy for treatment of colorectal and head and neck squamous cell carcinoma and (b) with external radiotherapy for treatment of head and neck squamous cell carcinoma. The conjugation of radionuclides to cetuximab in combination with the specific targeting properties of this antibody might increase its therapeutic efficiency. This review article gives an overview of the preclinical studies that have been performed with radiolabeled cetuximab for imaging and/or treatment of different tumor models. A particularly promising approach seems to be the treatment with therapeutic radionuclide-labeled cetuximab in combination with external radiotherapy. Present data support an important impact of the tumor micromilieu on treatment response that needs to be further validated in patients. Another important challenge is the reduction of nonspecific uptake of the radioactive substance in metabolic organs like liver and radiosensitive organs like bone marrow and kidneys. Overall, the integration of diagnosis, treatment and monitoring as a theranostic approach appears to be a promising strategy for improvement of individualized cancer treatment.

## 1. Introduction

Worldwide, cancer is one of the most common causes of death. In general, patients will be treated with approaches comprising surgery or external beam radiotherapy (EBRT) alone, or surgery combined with EBRT or chemotherapy, that have been developed and improved in the last years [1,2,3]. In EBRT usually 1.8 to 2 Gy fractions are delivered from a linear accelerator over several weeks. Radioimmunotherapy (RIT) approaches are applying radioactive antibodies (Ab) or Ab fragments in patients either locally close to the tumor or systemically with the goal to bind to tumor specific targets, thereby inactivating cancer cells. Remarkably, the curative treatment of metastases by RIT might be a special chance of this method. For patients with advanced inoperable stages of cancer, particularly head and neck cancer, primary radiochemotherapy still offers curative potential that has increased over the last decades by improvement of techniques and combined radiochemotherapy treatment approaches. However, currently around 50%–70% of all patients with advanced head and neck squamous cell carcinoma (HNSCC) develop locoregional recurrences after primary radiochemotherapy [4,5]. Thus, it is of high importance to develop and prove novel therapeutic strategies that could improve locoregional tumor control. Currently, successful targeted approaches for cancer therapy focus on receptors located on the surface of cancer cells that are higher expressed in cancer than in normal tissue.

Over many years the epidermal growth factor receptor (EGFR) has been investigated as a major target for the treatment of uncontrolled tumor growth. The EGFR, a glycosylated transmembrane protein, one of four members of closely related receptor tyrosine kinases (EGFR = ErbB1/HER1; ErbB2/HER2; ErbB3/HER3; ErbB4/HER4), is involved in regulating cell growth, differentiation and survival of cells. It is composed of an extracellular ligand binding region, a transmembrane region and an intracellular tyrosine kinase domain. The cytosine-rich extracellular domain binds endogenous growth factors, like epidermal growth factor (EGF), transforming growth factor alpha (TGF-α) [6], heparin-binding growth factor [7], amphiregulin [8] and betacellulin [9]. Binding of one of the endogenous ligands results in the formation of receptor homodimers (EGFR-EGFR) or receptor heterodimers (EGFR—homolog ErbB receptor) [10]. Dimerization causes autophoshorylation of the tyrosine residues that in turn initiates activation of signaling cascades. One of the main downstream signaling pathways is the MAP kinase system [11]. Activation of the MAPKs via Ras is regulating transcription of molecules for cell proliferation, migration, adhesion and survival [12]. Another major target, the PI3K/Akt signaling pathway, is involved in control of biological processes like growth, proliferation, angiogenesis, senescence, apoptosis, and formations of genetic aberrations [13]. Furthermore, of particular importance is the signal transduction pathway JAK/STAT, that mediates motility, invasion, adhesion, immune tolerance, cell survival and also proliferation [14,15].

The EGFR is often overexpressed in human malignancies such as HNSCC, gastrointestinal and abdominal carcinomas, lung carcinomas, carcinomas of the reproductive tract, melanomas, glioblastomas and thyroid carcinomas [16]. Although data are heterogeneous, overexpression is often associated with an aggressive tumor phenotype and a poor clinical prognosis. To target tumor cell proliferation or growth via EGFR, monoclonal antibodies (mAb) against this receptor have been developed. A promising potential therapeutic possesses the chimeric human-murine IgG1 mAb cetuximab (C225; Erbitux^®^, ImClone LLC), that has been approved by the Food and Drug Administration (FDA) for treatment of colorectal cancer as single drug or in combination with chemotherapy and of HNSCC in combination with radiation therapy or as monotherapy after failure of platinum-based therapy (2004 approval). Cetuximab, a 152 kDa molecule, is composed of two 449-amino-acid heavy chains and of two 214-amino-acid light chains interfaced both by covalent (disulfide) and non-covalent bonds [17]. The competitive binding of the mAb at the extracellular domain of the EGFR prevents binding of the natural ligands. On the other hand, cetuximab binding to EGFR also leads to receptor dimerization and internalization of the antibody-receptor-complex [18], not necessarily causing downregulation of membraneous EGFR expression [19]. Furthermore, cetuximab can induce antibody-dependent cell-mediated cytotoxicity [20].

The affinity of cetuximab toward EGFR is about tenfold higher than that of the endogenous ligands EGF or α-TGF (cetuximab 0.1–0.2 nM *vs.* EGF, α-TGF 1–2 nM) [21,22]. Blocking of the EGFR also affects the cell cycle by inducing upregulation of the cell cycle inhibitor p27Kip1. Consequently, EGFR expressing cells remain in a G1 arrest, preventing DNA synthesis [23,24,25]. Inhibition of tumor growth with cetuximab has in many cases been confirmed *in vivo* [18,26].

There are several studies about treatment of, particularly, head and neck cancer or colorectal cancer with cetuximab combined with chemotherapy, that show prolonged median overall survival [27,28,29], whereas similar treatment of non-small cell lung cancer remained uncertain and was not recommended [30]. Similarly, cetuximab paired with various chemotherapeutic regimens and/or other biological agents failed to improve the outcome of patients with pancreatic cancer [31].

## 2. Cetuximab Combined with Radiotherapy

In a clinical phase III randomized trial the combination of cetuximab and radiotherapy significantly improved locoregional recurrence and overall survival compared to radiotherapy alone for patients with locoregionally advanced HNSCC. The five years survival rate for treatment with cetuximab combined with radiation was 45.6% compared to 36.4% after radiation treatment alone [32]. However, also simultaneous radiochemotherapy improves survival compared to radiotherapy alone to a similar extent (33.7% *vs.* 27.2%) [4], and a direct comparison has never been performed prospectively. Thus, radiotherapy combined with cetuximab can be seen as alternative treatment option for specific cases but seems not superior to standard radiochemotherapy [33,34,35]. Some studies showed moderate improvements of local control and long-term survival after treatment with cetuximab plus radiotherapy [36,37]. Results of triple combination in randomized trials have preliminarily been reported and do also not support superiority over radiochemotherapy [38,39]. Concerning toxicity, combination of radiotherapy with cetuximab induces higher rates of mucositis, skin reactions and anaphylactic reactions, whereas radiochemotherapy leads to nephrotoxicity and myelosuppression [40].

To improve treatment outcome by pre-selection of patient subgroups that are expected to benefit from combined radiotherapy and cetuximab, mechanistic as well as functional pre-clinical *in vivo* studies are essential. In different HNSCC models simultaneous radiotherapy and cetuximab leads to heterogeneous effects on local tumor control, potentially correlating with genetic EGFR amplification [41] but not with EGFR expression [42]. Further, potential reasons for cetuximab resistance include the most frequently detected EGFR mutation class III variant (EGFR**v**III) [43], or mutation of the EGFR tyrosine kinase domain [44], or mutation of the oncogene KRAS, BRAF or NRAS that can activate the EGFR even during EGFR inhibition [45,46,47]. However, these molecular features are rare or not existent in head and neck squamous cell carcinoma, so that the mechanisms of the functional heterogeneity of tumor response are still not well understood.

Recently, the combination of targeted diagnostic and therapeutic applications (theranostics) is developing. The corresponding noninvasive imaging methods like SPECT or PET are appropriate methods to characterize the status of EGFR expressing tissue [48]. According to the application appropriate radionuclides are required. Since the majority of applied radionuclides are metals (Table 1), a rather extensive chelation chemistry has been developed to couple them to mAbs like cetuximab.

## 3. Radiolabeled Cetuximab

In order to estimate the status of EGFR expression, cetuximab was labeled with different radionuclides. Since EGFR is overexpressed in a variety of tumors, the accumulation of radiolabeled cetuximab in the tumor cells could serve as complementary diagnostic tool. Table 1 summarizes diagnostic and therapeutic radionuclides used for labeling of cetuximab conjugates. 

Due to the size of the mAb its pharmacokinetics is slow with a biological half-life of 63 to 230 h [51]. The biological half-life of a substance in a biological organism is exclusively mediated by biological processes and represents the time during which the amount of the respective substance decreases to half of its original value. Moreover, of special importance is the effective half-life, the time during which the amount of a radiopharmaceutical is decreased to half of its value; that is altogether determined by the combination of the biological half-life of the substance and the physical half-life of the used radionuclide. The effective half-life for radiopharmaceuticals is predominantly influenced by the physical half-life of the radionuclide, which is suitable either for imaging or therapy. Accordingly radiolabeled cetuximab requires longer-lived radionuclides for monitoring. Furthermore, immunogenicity for diagnosis is unwanted, but concentrations of radiolabeled cetuximab with high specific activity are usually below nanomolarity (picomolar level) and do not show physiological effects.

**Table 1:** Diagnostic and therapeutic radionuclides for labeling of cetuximab conjugates ^a^.

Radionuclide	Half-life	Main types of decay (probability) ^b^	E_max_ (MeV)	Production
Radionuclides for imaging				
^64^Cu	12.7 h	β^+^ (17.5%)	0.653	cyclotron
		β^−^ (38.5%)	0.579	^64^Ni(p,n)^64^Cu
		EC (43.5%)	1.675	
^68^Ga	1.13 h	β^+^ (87.7%)	1.899	^68^Ge/^68^Ga generator
		EC (8.9%)	2.921	
		γ (3.2%)	1.077	
^86^Y^c^	14.7 h	β^+^ (11.9/5.6%)	1.221/1.545	cyclotron
		γ (83/32.6%)	1.077/0.628	^86^Sr(p,n)^86^Y
^89^Zr^c^	3.3 dβ^+^ (22.7%)		0.902	cyclotron
		γ(100%)	0.909	^89^Y(p,n)^89^Zr
^99m^Tc	6 h	γ (99%)	0.141	^99^Mo/^99m^Tc generator
^111^In	2.8 d	γ (100%)	0.245	cyclotron
		EC (99.99%)	0.417	^111^Cd(p,n)^111^In
^124^I^c^	4.2 d	β^+^ (11.7/10.8%)	1.535/2.135	cyclotron
		γ (63/10.9%)	0.603/1.691	^124^Te(p,n)^124^I
^125^I	59.4 d	γ (100%)	0.035	nuclear reactor
		EC (100%)	0.150	^124^Xe(n,γ)^125^Xe→^125^I
^90^Y	2.67 d	β^− ^(99.98%)	2.279	^90^Sr/^90^Y generator
^131^I	8 d	β^− ^(89.4/7.4%)	0.606/0.334	nuclear reactor
		γ (83.1/7.3%)	0.364/0.637	^130^Te(n,γ)^131^Te→^131^I
^177^Lu	6.65 d	β^−^ (79.3/11.6%)	0.498/0.177	nuclear reactor
		γ (20.3/11%)	0.113/0.208	^176^Yb(n,γ)^177^Yb→^177^Lu
^213^Bi	45.6 min	α (1.9%)	5.981	^225^Ac/^213^Bi generator
		β^−^ (66.2/30.8%)	1.423/0.983	

^a^ data from LNHB: http://www.nucleide.org/DDEP_WG/DDEPdata.htm [49]; ^b^ specification of the main transitions; ^c^ data from Lubberink Herzog 2011 [50]; EC electron capture; IC internal conversion.

### 3.1. Radionuclides

The selection of appropriate radionuclides with regard to their requested application is a crucial issue. It is necessary to consider different characteristics of radiation according to the requirements, like decay characteristics, particle range and physical half-life of the radionuclide. Anyhow, radionuclide selection is often done in terms of economic aspects [52]. The preferred approach for treatment of bulky tumors is the application of beta-emitting radionuclides and in future even of alpha emitters. For eradication of small clusters of cancer cells radionuclides that emit auger electrons are considered to be advantageous [53].

#### 3.1.1. Radionuclides for C225 Conjugates Used as Imaging Probes

**^64^Cu**. As important positron-emitting radionuclide ^64^Cu has the potential for application in diagnostic imaging and, to some extent, also for targeted radiotherapy [54] because it additionally emits β^−^ particles. By the nuclear (p,n) reaction on enriched ^64^Ni high specific activity of ^64^Cu can be achieved [55,56] to apply for labeling of biomolecules. Since the positron-energy of ^64^Cu is rather low, comparable with that of ^18^F (0.633 MeV), ^64^Cu PET images exhibit good resolution of high quality. Copper per se is participating in certain metabolic processes like binding on a series of enzymes such as superoxide dismutase, cytochrome c oxidase, or dopamine hydroxylase [57]. Therefore, copper ions should form complexes with high kinetic and thermodynamic stability. Cu(II) is forming stable chelate complexes, thus, in the last years there has been an active research development in this area. In particular, the chelators 1,4,7,10-tetraazacyclododecane-1,4,7,10-tetraacetic acid (DOTA), 1,4,8,11-tetraazacyclotetradecan-1,4,8,11-tetraacetic acid (TETA) and 1,4,7-triazacyclononane-1,4,7-triacetic acid (NOTA) have been studied. ^64^Cu-TETA complexes are more stable than ^64^Cu-DOTA complexes, however, it has been shown, that ^64^Cu-TETA-octreotide is subjected to transchelation [58]. Several studies have been published with ^64^Cu-labeled cetuximab using exclusively the DOTA chelator [45,59,60,61,62]. Recently, investigations of a mAb conjugated both with DOTA and NOTA, and labeled with ^64^Cu, suggested that the NOTA conjugate was superior to the DOTA conjugate by showing better *in vivo* stability [63].

**^68^Ga**. ^68^Ga is a short-lived positron emitter and can be easily and relatively cheap generated with a ^68^Ge/^68^Ga generator. Similar to ^64^Cu ^68^Ga forms stable complexes with DOTA and NOTA. The label of ^68^Ga is more appropriate for smaller molecules with faster biokinetics and bioavailability than for mAb with the aim to diagnose and localize tumors. To target the EGFR ^68^Ga-labeled peptides [64], Fab fragments [65], affibodies [66] or nanobodies [67] have been applied. ^68^Ga might also be applied in pretargeting approaches, where conjugates of e.g., hapten peptide [68,69], oligonucleotide [70] or peptide nucleic acid [71], after achieving high accumulation in the target tissue, would bind the ^68^Ga-labeled complementary parts. Furthermore, ^68^Ga can be replaced with the gamma emitter ^67^Ga having a longer physical half-life of 3.26 d, appropriate for SPECT, and thus can be applied for investigations on longer circulating biomolecules like antibodies. In the study of Engle *et al.* [72] the positron emitter ^66^Ga with a half-life of 9.4 h could be achieved with sufficient specific activity and was recommended as surrogate for ^68^Ga or ^67^Ga. Exploiting the longer half-life compared to ^68^Ga, ^66^Ga as label for NOTA-cetuximab was investigated in breast tumor bearing mice. However, the resolution of the images due to high energy positrons as well as accumulation in the tumor appeared to be not optimal.

**^86^Y**. ^86^Y is a positron emitter generally produced via the nuclear (p,n) reaction from enriched [86Sr]SrCO_3_ [73]. ^86^Y/^90^Y (and ^177^Lu) form a matched-pair, thus the same chelators can be used. However, the half-life, presumably good for imaging of smaller molecules like mAb fragments and peptides, seems, similar to ^64^Cu, short for imaging of large mAbs, and, compared to ^89^Zr, also short concerning logistic aspects like transport [74]. Furthermore, ^86^Y emits high energy γ-photons, which together with the annihilation photons might result in false coincidences and thus in quantification artifacts [75,76] affecting the spatial resolution and imaging quality [77]. But emitting positrons abundantly, almost twice as much as ^64^Cu, the activity of ^86^Y required for quantitative immuno-PET can be kept rather low. Anyhow, with ^86^Y promising PET studies using tumor mouse models have been performed [78,79,80,81,82] among others also with cetuximab, that was conjugated to the bifunctional chelator (BFC) CHX-A′′-DTPA [83,84] under mild conditions [85]. As PET/RIT pair ^86^Y as surrogate for ^90^Y seems to be convenient. It might to be more suitable than the pair ^89^Zr/^90^Y since the uptake of ^89^Zr-labeled cetuximab particular in bone was higher than that of ^88^Y-labeled cetuximab (^88^Y as surrogate for ^90^Y) [74]. Also the PET/RIT surrogate pair ^86^Y and ^177^Lu can be of interest.

**^89^Zr**. ^89^Zr is a long-lived positron emitter. The production of choice is the (p,n) reaction on ^89^Y, an element that does not require enrichment due to its natural abundance of 100% [86]. Since cetuximab has a rather long biological half-live of 63 to 230 h [87] ^89^Zr is an appropriate radionuclide for application in so-called immuno-PET and offers high sensitivity, resolution and precise quantification. Although it emits also γ photons those do not interfere with the PET image quality and accurate quantification [88]. In PET ^89^Zr might be used as surrogate to predict biodistribution and dosimetry of ^177^Lu- and ^90^Y-labeled mAb conjugates [74,89]. It is coupled to cetuximab via N-succinyl desferrioxamine B. Biodistribution was comparable with that of ^86^Y- and ^177^Lu-radiolabeled cetuximab conjugates. Differences can occur due to coupling with other chelators that change the pharmacokinetics and *in vivo* stability [74], but basically ^89^Zr and ^90^Y as well as ^89^Zr and ^177^Lu appear to be good PET/RIT pairs.

**^99m^Tc**. Since the gamma emitter ^99m^Tc has favorable physical properties for scintigraphic imaging and can be produced with low costs by the ^99^Mo/^99m^Tc generator, this radionuclide has been used widely for labeling of radiopharmaceuticals. As stable complex with ethylenedicysteine [90] a conjugate to cetuximab has been formulated [91]. The uptake of this cetuximab conjugate in tumor tissue was still higher than the uptake of ^99m^Tc complex only, but not convincingly high for analyzable imaging to achieve. Besides, an unexpected high kidney uptake was observed in human breast tumor-bearing rats [92]. The half-life of 6 h for ^99m^Tc is too short for imaging of mAb like cetuximab when the highest accumulation of the antibody in the tumor is expected after 2 to 3 days. Moreover, in patient studies to visualize head and neck cancer correlations of the imaging results with clinical findings are missing. Furthermore, high liver uptake was observed compared with an uptake in HNSCC [92] that was not sufficiently high. As already discussed for ^68^Ga Ab conjugates with ^99m^Tc are not convenient for *in vivo* applications. It would be more adequate to couple ^99m^Tc complexes to smaller molecules which reach their target faster than mAbs.

**^111^In**. ^111^In is a cyclotron-produced radiometal, and one of the most commonly used radionuclides for SPECT [93] especially as label for mAb due to its adequate physical half-life (2.8 d). Even it emits Auger and internal conversion electrons with low energy that might be interesting for therapeutic approaches [94], primarily γ radiation is used for diagnostic imaging. ^111^In-labeled mAb conjugates with the chelators DOTA or DTPA have been investigated in small animals [85,95,96] and human [97,98].

**^124^I**. ^124^I is a positron emitter with a complex decay scheme [99]. In addition to two positron emitting transitions, ^124^I emits γ rays at more than 90 transitions resulting in increased random coincidences in PET. The so-called true-coincidence γ ray background may disturb the PET imaging. And the high energy of the emitted positrons from ^124^I also contributes to a declined resolution. Furthermore, iodine has been described for the tendency to separate from mAb after injection, because of metabolic degradation, and so it might accumulate in different organs and the interpretation of PET images turns out to be difficult [100]. However, radioiodine can be used for direct labeling without a chelator. In many cases this facilitates the labeling of biomolecules. In this regard, ^124^I has been used as an imaging nuclide surrogate for ^131^I [101]. Moreover, the relatively long half-life justifies the use of ^124^I-labeled mAbs [102,103]. Recently an anti-EGFR antibody has been ^124^I-labeled and studied successfully *in vitro* and *in vivo* in mice bearing glioma xenografts [104].

**^125^I**. Since ^125^I has a long half-life of 60 d and emits low-energy γ radiation, it can be detected by a gamma-counter. This radionuclide is often coupled to antibodies for application in radioimmunoassays (RIA), and also in preclinical EGFR investigations [105,106,107]. Furthermore, ^125^I-labeled cetuximab was applied in tumor bearing mice and showed in general lower uptake in the tumor compared with radiometal labeled cetuximab [74,108,109]. Due to the long half-life and its tendency to degrade faster than radiometal-antibody-conjugates ^125^I will not be introduced as label for antibodies in clinical trials.

#### 3.1.2. Radionuclides for Cetuximab Conjugates Used as Therapeutics

**^90^Y**. The therapeutic β^−^ emitter ^90^Y is of particular interest for medical applications due to its suitability for irradiating primary tumor lesions. It is available at moderate costs via ^90^Sr/^90^Y-generators, has an appropriate half-life for RIT and a high β^−^ emission energy with a tissue penetration range of up to 12 mm [110]. Thus, it is more suitable for RIT of large bulky solid tumors. Caution and good biodosimetry is necessary when a tumor is located adjacent to critical organs, especially if combined with EBRT. Yttrium in general should be applied within a stable chelate complex since in free condition it deposits in the bone [111]. The absence of γ emission by ^90^Y makes it not trivial for *in vivo* imaging [112,113]. Alternatively, for ^90^Y the longer lived ^88^Y (half-life 106.6 d, β^+^ 0.2%, E_β-max_ 0.76 MeV; γ 99%, E_γ_ 1.836 MeV) has been used as substitute to estimate biochemical properties [74,114,115]. However, its γ energy is too high for imaging, and the low amount of positrons might be reasonable only for small animal PET to prevent significant scatter of the prompt γ rays into the PET energy window [116]. Recently, ^90^Y-labeled cetuximab conjugates have been applied in RIT [117], also in combination with external radiation [118,119] (see Section 3.5.).

**^131^I. **^131^I is a β^−^ emitter with concomitant γ radiation. The β^−^-radiation is used for internal radiotherapy of hyperthyroidism [120] and different tumor types, like neuroblastoma, pheochromocytoma [121], and thyroid cancer [122], whereby the γ radiation part is often applied for SPECT imaging [123]. A crucial advantage is the low cost of the radionuclide production, but a disadvantage the low stability resulting in corresponding deiodination reactions *in vivo*. Recently, ^131^I-labeled cetuximab treatment has been applied in combination with irradiation on epidermoid cancer cells (A431). In result the combination of ^131^I-cetuximab with external radiation inhibited cell proliferation *in vitro* [124].

**^177^Lu**. ^177^Lu is the more favorable therapeutic radionuclide for treatment of small tumors due to its low energy and tissue penetration of about 1.5 mm [110]. The physical half-life is sufficient for preparation, transport and delivery of therapeutic doses to tumors applied as immunoconjugates like mAbs. Due to its low energy γ-lines it is possible to perform imaging. The chemistry of ^177^Lu resembles the metallic radionuclide ^90^Y forming also stable complexes with DOTA and cysteine-based DTPA. Several ^177^Lu-labeled Ab conjugates have been studied [125,126,127], including cetuximab conjugates [74,109,128].

**^213^Bi**. Recently, mAb have also been labeled with α emitters like ^213^Bi that as therapeutic radionuclide might be more efficient in killing tumor cells with less damage in the surrounded healthy tissue in targeted therapy [129,130]. The production with a ^225^Ac/^213^Bi generator has been developed even for application in clinical use [131]. The range of α-particles is rather short in tissue in comparison to beta particles (50–80 µm *vs.* 0.8–12 mm); they have a much higher linear energy transfer (100 keV/µm *vs.* 0.2 keV/µm) [129]. Currently, there are clinical trials for different types of cancer with targeted high potent α-emitters [132,133,134]. In an *in vitro* study ^213^Bi-labeled CHX-A′′DTPA-cetuximab showed effective double-strand breaks on different human breast cancer cells, but for an approach in patients the safety of targeted α-emitter-labeled radioconjugates has to be evaluated [135]. However, due to the short half-life of only 46 min an application of ^213^Bi-labeled to CHX-A′′DTPA-cetuximab would be rather questionable and likely will not enter the clinics.

### 3.2. Linking Chelating Units

**Figure 1:**
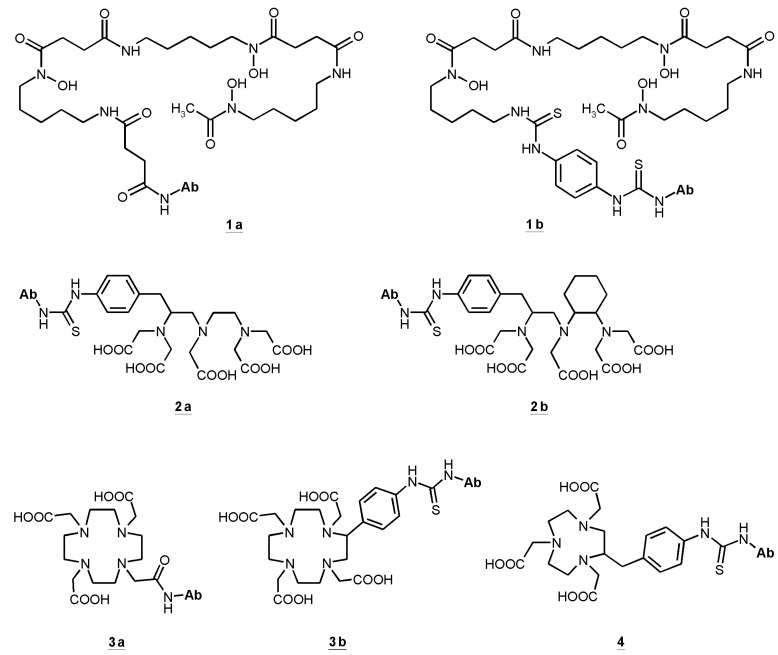
Bifunctional chelators (BFC) used in cetuximab conjugates: succinylated desferoxamine (*N*-sucDf, **1a**), desferoxamine-p-SCN (Df–Bz–NCS, **1b**), p-SCN-Bn-DTPA (**2a**), CHX-A′′-DTPA (**2b**), DOTA-NHS-ester (**3a**), p-SCN-Bn-DOTA (**3b**), p-SCN-Bn-NOTA (**4**).

Almost all radionuclides for diagnostics and therapy of different types of cancer coupled to antibodies, antibody fragments or peptides are radiometals. That requires chelation chemistry for the attachment to the ligands. Several chelators have been conjugated to cetuximab. Hereby it is necessary to find the balance between the required coupling conditions to obtain a stable conjugate preferably without degradation, loss of affinity and immunoreactivity. For stable coupling of radiometals to antibodies and preservation of their special features, mild conjugation procedures have to be established. The chelating agents of choice should form stable metal complexes as well as provide specific functional groups to enable the conjugation to a protein. Such bifunctional chelators (BFC) have to be characterized for several properties: thermodynamic and kinetic stability, pH-dependent dissociation and serum stability [136]. To determine *in vivo* stability of any labeled conjugate only suitable *in vivo* models can provide such information. Figure 1 illustrates the bifunctional chelators used in cetuximab conjugates.

One of the first used bifunctional chelating agents was desferrioxamine B that, conjugated to an antibody, has been radiolabeled with ^111^In [137]. Derivatives of desferrioxamine, originally developed as chelators for Fe(III), form stable complexes with In(III), Ga(III) and Zr(IV). Thus, desferrioxamine antibody conjugates labeled with ^67^Ga have earlier been investigated [138]. Recently, desferrioxamine derivatives were conjugated to mAb [86,139,140]. Labeled with ^89^Zr the conjugates showed promising results with regard to radiochemical purity, integrity, preservation of immunoreactivity and stability [86]. Moreover, with ^89^Zr-desferrioxamine-cetuximab-conjugates small animal PET studies revealed convincing results with good resolution showing high accumulation in different tumors [141,142]. Particularly, high uptake was demonstrated in FaDu tumors, a model for HNSCC [140].

Starting from DTPA several bifunctional derivatives have been developed and investigated [143,144]. Recently, CHX-A′′-DTPA (correct: *p*-SCN-Bn-CHX-A′′-DTPA) has been used for conjugation with antibodies to form sufficiently stable ^86^Y, ^90^Y, and ^111^In immunoconjugates, which could successfully be applied *in vivo* [83,145,146,147]. Among the backbone-substituted DTPA derivatives CHX-A′′-DTPA showed very good *in vitro* and *in vivo* stability [143,144] and it can be conjugated and radiolabeled under mild conditions to preserve the immunoreactivity of the resulting conjugate. 

The kinetic stability of a radiometal complex plays a more important role for *in vivo* stability than the thermodynamic stability [148], but still, possible predictions can be just assumed. For example the complex [^111^In-DOTA]^-^ is kinetically more stable than [^111^In-DTPA]^2−^, but thermodynamic stability of In(III)-DTPA is about 5 orders higher than the appropriate In(III)-DOTA complex [96]. 

It was shown that CHX-A′′-DTPA, conjugated to a HER2-specific affibody, provides better cellular retention of the radiolabeled Ab, better tumor accumulation and better tumor-to-organ dose ratios in comparison with DOTA [149]. DTPA antibody conjugates have a satisfactory labeling efficiency [150].

Bifunctional chelating units based on DOTA are the chelators of choice for yttrium isotopes and ^177^Lu [74,125]. DOTA derivatives are also often used for chelating ^64^Cu [59,151], although it has been claimed that DOTA is not the optimal chelator for ^64^Cu, because ^64^Cu-DOTA shows a certain instability *in vivo* [58,148]. However, stability constants measured in an *in vitro* chemical system [152] cannot represent *in vivo* conditions. For instance, it has been described that transchelation for a ^64^Cu-DOTA antibody was much higher than for the same ^64^Cu-NOTA antibody [63]. Cross-bridged macrocycles show greater stability with ^64^Cu. However, there is the need for harsh labeling conditions (95 °C for 2 h) [153] which are incompatible for protein labeling. But still, the tumor uptake of ^64^Cu-DOTA-cetuximab is relatively high [59,60,61,117,151].

Particular importance has attained the preservation of the immunoreactivity of the antibody after conjugation reactions. A flow cytometry study showed still high binding capacity after conjugation of CHX-A′′-DTPA to cetuximab [154]. Other studies present a preserved immunoreactivity [83,85], and the high affinity of cetuximab to EGFR was kept [119].

The uptake of radiolabeled cetuximab in EGFR expressing model tumors in mice was, in general, significantly higher compared with the uptake in the main body parts, except the liver (Table 2). The decline from the blood appeared to be faster than from the tumor and, unfortunately, also from the liver. Anyhow, the outcome was a high tumor-to-muscle or tumor-to-background ratio. Of note, the data were comparable for most conjugates applied, excepting those using non-appropriate chelating units [91] or those using non-appropriate radionuclides [72]. These are data from rodent models naturally not expressing human EGFR. Therefore, an extrapolation of the biodistribution to human pharmacological characteristics might be difficult, reflecting not the true relations (see below).

**Table 2:** Radiolabeled cetuximab conjugates studied in tumor-bearing mice.

Radionuclide	Chelator	Tumor type	Application	Tumor uptake	Tumor/muscle ratio	Liver uptake	Reference
				(%ID/g, 24 h post-injection)			
^64^Cu	DOTA	h GB	i.v.	12.5	5	15	[59]
		h PC		11	4.5	6 (rat)	
		h CRC		~5		2	
		m CRC		10		4	
		h M					
^64^Cu	DOTA	h CC	i.v.	14	3.5	16	[60]
^64^Cu	DOTA	PC-3	i.v.	15	15	17	[151]
^64^Cu	DOTA	A431	i.v.	18.5	8.5	13	[61]
		h M		2.6	1.3	10	
^64^Cu	DOTA	h HNSCC (UMSCC22B)	i.v.	19	6	11	[117] ^a^
^64^Cu	DOTA	h HNSCC (UMSCC1)	i.v.	6	2.5	13	[117] ^a^
^64^Cu	NOTA	m BC	i.v.	4	4	19	[155]
^64^Cu	NOTA	m BC	i.v.	20	10	19	[54]
^66^Ga	NOTA	h BC	i.v.	4	5	6	[72] ^b^
^86^Y	DTPA	h CRC	i.v.	21	11	10	[83]
^88^Y	DTPA	A431	i.p.	21	14	11	[74]
^88^Y	DOTA	A431	i.p.	17	11	10	[74]
^89^Zr	Df	h GB	i.v.	15	15	10-12	[141]
		h CRC		10		10	
		A431		8		8	
		h BC		3		3	
^89^Zr	Df	A431	i.v.	3.5^c^	10^d^	11^c^	[142]
^89^Zr	Df	A431	i.p.	21	17	10	[74]
^89^Zr	Df	A431	i.v.	15	8	9	[139]
^89^Zr +	Df	A431	i.v.	22	19	20	[156]
^89^Zr + ½ dye ^e^	Df			20	19	22	
^89^Zr + 1 dye	Df			20	19	25	
^89^Zr + 2 dye	Df			13	16	40	
^99m^Tc	EC	h BC	i.v.	0.3	8.5	0.6	[91]
^86^Y	DTPA	h CRC	i.v.	21	11	10	[83]
^88^Y	DTPA	A431	i.p.	21	14	11	[74]
^88^Y	DOTA	A431	i.p.	17	11	10	[74]
^90^Y	DOTA	normal rats	i.v.			2	[157]
^177^Lu	DOTA	A431	i.p.	18	12	13	[109]
^177^Lu	DOTA	A431	i.p.	17.5	12	8-13	[74]
^177^Lu	DTPA	A431	i.p.	17.5	12	7	[74]
^111^In	DTPA	A431	i.v.	11	29	47	[158]^ f^
	DTPA-PEG	A431		8.7	13	25	
^111^In	DTPA	h OC	i.v.	8.8	11	4	[95]^ f^
^111^In	DTPA	h CRC	i.v.	28/24^g^	28/24^g^	9/16^g^	[85]
		h PC		16	16	6	
		h PancC		10	10	10	
		h OC		13	13	10	
		h M		3	3	9	
^111^In	DTPA	h HNSCC	i.v.	20	14	11	[108]
^111^In	DTPA	h BC	i.v.	18/40^f^	13	11/15^f^	[135]
^111^In	DTPA	h HNSCC (FaDu)	i.v.	27	13	8	[159]
^125^I		h HNSCC	i.v.	11	8	7	[108]
^125^I		A431	i.p.	8.4	5.6	4	[109]
^125^I		A431	i.p.	8	5	4	[74]

Df desferrioxamine chelating unit; EC ethylenedicysteine; h human; m murine; GB glioblastoma; CRC colorectal carcinoma; A431 human epidermoid carcinoma; BC breast carcinoma; PC prostate carcinoma; CRC colorectal carcinoma; CC cervical cancer; M melanoma (MDA-MB-435); HNSCC head and neck squamous cell carcinoma; UMSCC22B cells of the lymph node the oropharynx; UMSCC1 cells of the oral cavity; OC ovarian carcinoma; PancC pancreas carcinoma; FaDu hypopharyngeal carcinoma cell line; ^a^ 20 h after radiotracer injection; ^b^ 36 h after radiotracer injection; ^c^ %ID/mL tumor PET analysis; ^d^ tumor to background (pelvic); ^e^ different equivalents of the dye IRDye800CW; ^f^ 48 h after radiotracer injection; ^g^ value from two different types of h CRC xenografts.

### 3.3. Liver Accumulation

Liver accumulation appears to be a general problem using mAb-based immunoimaging and immunotherapeutics in animal studies, Table 2. Overall, the liver uptake of ^90^Y-DOTA- and ^64^Cu-DOTA cetuximab in rats appears to be proportionally lower as compared to mice. Biodistribution studies revealed that cetuximab is eliminated partly via the reticuloendothelial system, binding on fc receptors of lymphocytes, macrophages *etc*. passing sinusoid capillaries especially into the liver. Thus, a considerable part is accumulating in this organ. Table 2 shows only the values for 24 h post injection, since the accumulation in the liver did not increase after 24 h and declined only slowly thereafter, whereas the highest tumor uptake of a cetuximab conjugate was measured after 72 h. In an ^89^Zr-labeled cetuximab study a multimodal imaging approach was investigated where a dye, emitting fluorescence in the near-infrared region, was conjugated additionally to the Ab [156]. In this study, the more dye units the Ab received the lower was the tumor uptake and the higher the liver uptake.

In general, accumulation of ^125^I-labeled cetuximab in the liver was lower compared to the radiometal-labeled cetuximab studies, but also in tumor this conjugate accumulated considerably less. Thus, the question arises to what extent metal chelate complexes influence the uptake of the conjugates. As noted above, radioiodine labeled antibodies are subject to degradation and deiodination due to their *in vivo* instability to proteolysis. A faster degradation of iodine labeled antibodies after internalization causes faster clearance from the target cells and results in images with lower tumor contrast, thus not reflecting the real distribution of the Ab [160]. Moreover, the risk of radioiodine accumulation in the thyroid contributes to the inappropriateness of radioiodine as a therapeutic tracer outside the thyroid.

A dimension independent from the weight of the organs and the body weight is the standard uptake value (SUV). Considering the SUV of 1.6 for liver and 4.2 for FaDu tumor in the biodistribution with ^90^Y-CHX-A′′-DTPA-cetuximab, liver accumulation appears to be justifiable. PET studies with ^86^Y-CHX-A′′-DTPA-cetuximab using FaDu bearing mice showed similar accumulation distribution [83]. With ^111^In-labeled CHX-A′′-DTPA-cetuximab in tumor bearing mice higher liver uptake was observed [85,108,158]. The liver accumulation might partly be due to nonspecific uptake of labeled yttrium caused by transchelation [161]. Since applications with radiolabeled mAbs have been limited by liver uptake, an approach for reduction was the modification of the conjugate with PEG, or also the pretreatment with cetuximab [158]. Recently, the biodistribution of ^111^In-DTPA-cetuximab-fragments have been compared with ^111^In-DTPA-cetuximab in FaDu tumor bearing mice. In this study the fragments showed significantly lower uptake in the tumor, but lower uptake in the liver could not be observed, it was even higher within the first 4 h after administration. In addition, more radioactivity was measured in the kidney [159]. In a first clinical imaging study in patients with lung squamous cell carcinoma ^111^In-DTPA-MAb225, the murine forerunner of cetuximab, also showed a high liver accumulation [98]. The uptake of DTPA-cetuximab conjugates in the liver appears to be somewhat lower compared to the liver uptake of ^64^Cu-DOTA-cetuximab. ^89^Zr-desferrioxamine-cetuximab conjugates revealed a similar liver uptake as the yttrium-labeled conjugates. Histological assays of liver tissue have been performed after RIT, whereby no changes were observed [118]. However, a conclusion on potential normal organ toxicity is restricted to the experimental animals [162], because the antibody cetuximab is specific to human EGFR and thus is expected to show a differential organ distribution in humans compared to animals.

Specifically for cetuximab no binding to EGFR in frozen liver sections of mice and rats could be detected, whereas strong cross-reactivity was observed with EGFR expressed on the cell surface of various types of human tissue including skin, lung, and liver [163]. Thus, it is important to consider that the elimination of cetuximab, also in the radiolabeled state, needs to be evaluated separately in humans and cannot be extrapolated from rodent studies.

### 3.4. The Enhanced Permeability and Retention Effect

Non-specific tumor uptake of radiolabeled Ab often is caused by the enhanced permeability and retention (EPR) effect. Aberrant defective membrane formations of tumor blood vessels with wide fenestrations are leading to an enhanced vascular permeability. Besides, a malfunction of lymphatic vessels in tumor tissue impairs the clearance of macromolecules and lipids, so that they remain in the tumor interstitium for longer time. The EPR effect [164,165] has also been described when labeled antibodies have been used in studies for tumor diagnostics or treatment [166,167]. In a phase I imaging trial with ^111^In-labeled murine DTPA-MAb225 in patients with lung cancer several patients received the isotope-matched ^111^In-labeled control mAb [98]. Presumably, due to the EPR effect half of this control group did not show significant differences to the specific Ab. A number of studies used this labeled isotype of IgG1 as negative control to determine nonspecific tumor uptake [54,151,168,169]. Vascular endothelium in tumors also can be perturbed by hypoxic areas [170]. Thus, beside aberrant vessels in the tumor tissue also hypoxia seems to contribute to the EPR effect.

### 3.5. Therapeutic Approaches with Labeled Cetuximab

Combination of radioimmunotherapeutic approaches, e.g., radiolabeled cetuximab, with curatively intended radio(chemo)therapy are a promising research strategy to improve locoregional tumor control in head and neck or other cancer entities. The therapeutic success depends on the radioligand concentration in the tumor which, in addition to the target expression, seems to depend on tumor microenvironmental parameters [118].

After binding to the EGFR, radiolabeled cetuximab internalizes into the cell [60,107,108] and can cause there, additional damage of the cell as well as to neighbor cells. The combination of ^90^Y-labeled cetuximab (^90^Y-CHX-A′′-DTPA) treatment with subsequent irradiation reduced clonogenic cell survival more compared to external irradiation alone [119]. Unlabeled cetuximab caused a radiosensitizing effect [171,172] in one out of three cell lines [119]. Recently, an EGFR expression depending number of DNA double strand breaks (DSB) caused by ^90^Y-CHX-A′′-DTPA was demonstrated *in vitro* [173]. Furthermore, also in different breast cancer cells, which were sensitized with an inhibitor of DNA-dependent-protein-kinase, DNA DSB have been assessed after treatment of ^213^Bi-labeled cetuximab [135]. Rades *et al.* [124] showed that the highest antiproliferative effect in epidermoid cancer cells (A431) occurred after combined treatment with therapeutic ^131^I-labeled cetuximab and irradiation. These data suggest that higher radiation dose promotes and increases the uptake of the radiolabeled conjugate into the tumor cells.

*In vivo*, three different human squamous cell carcinoma models have been evaluated in nude mice. Two to 4 days after external beam single dose irradiation either unlabeled or ^90^Y-labeled cetuximab was applied. While one out of three tumor models did not respond, tumor growth delay could significantly be prolonged in two other HNSCC xenograft models [118]. Permanent local tumor control was evaluated for the non-responder and one responder-model, confirming non-response in UT-SCC5 but a significant improvement of local tumor control in FaDu, the latter being a non- or minimal-responder to radiotherapy with unlabeled cetuximab [41,118,174]. A combined parameter on tumor micromilieu, specifically perfusion, and EGFR expression, appeared as a candidate biomarker for tumor response—this parameter can be measured using PET imaging with ^86^Y-cetuximab as a tracer. In another study colon tumor-bearing mice showed higher survival after treatment combination of the cytostatic drug cisplatin followed by ^64^Cu-DOTA-cetuximab and suggested a role of the tumor suppressor protein p53 for the transport of ^64^Cu into the cell nucleus [175]. Here, even a KRAS mutated cetuximab-resistant tumor model was responding [54]. In a recent case report a patient with brain metastases from non-small cell lung cancer was treated with low concentrations of ^131^I-labeled cetuximab in addition to therapeutic cetuximab treatment and whole brain irradiation. SPECT was applied to monitor the treatment and showed accumulation of ^131^I-labeled cetuximab in the brain metastases which showed a decrease in size during the treatment. It remains to be elucidated whether cetuximab generally passes the blood-brain-barrier or only in specific patients [176].

## 4. Conclusions

Radiolabeled cetuximab derivatives in combination with external radiotherapy or established chemotherapy appear to be a promising theranostic approach for treatment of epithelial tumors, thus fostering more individualized treatment strategies. Since considerable heterogeneity of the functional response to labeled or unlabeled targeted treatments is obvious also within one histological tumor type, there is a clear need to establish predictive biomarkers for the curative effect of such treatments. One good candidate that needs to be validated in patients is PET imaging using labeled cetuximab as a diagnostic tracer. So far, treatment of patients with therapeutic radionuclide-labeled cetuximab has not yet entered into the clinics. Important open questions include the distribution and accumulation of the tracer in healthy organs in humans as well as the feasibility of the combined high-dose EBRT and RIT approach in patients.

## References

[1] Gunderson L.L., Ashman J.B., Haddock M.G., Petersen I.A., Moss A., Heppell J., Gray R.J., Pockaj B.A., Nelson H., Beauchamp C. (2013). Integration of radiation oncology with surgery as combined-modality treatment. Surg. Oncol. Clin. N. Am..

[2] Galaal K., van der Heijden E., Godfrey K., Naik R., Kucukmetin A., Bryant A., Das N., Lopes A.D. (2013). Adjuvant radiotherapy and/or chemotherapy after surgery for uterine carcinosarcoma. Cochrane Database Syst. Rev..

[3] Yang H., Diao L.Q., Shi M., Ma R., Wang J.H., Li J.P., Xiao F., Xue Y., Xu M., Zhou B. (2013). Efficacy of intensity-modulated radiotherapy combined with chemotherapy or surgery in locally advanced squamous cell carcinoma of the head-and-neck. Biologics.

[4] Pignon J.P., le Maître A., Maillard E., Bourhis J., MACH-NC Collaborative Group (2009). Meta-analysis of chemotherapy in head and neck cancer (MACH-NC): An update on 93 randomised trials and 17,346 patients. Radiother. Oncol..

[5] Bourhis J., Overgaard J., Audry H., Ang K.K., Saunders M., Bernier J., Horiot J.C., le Maître A., Pajak T.F., Poulsen M.G. (2006). Meta-Analysis of Radiotherapy in Carcinomas of Head and neck (MARCH) Hyperfractionated or accelerated radiotherapy in head and neck cancer: A meta-analysis. Lancet.

[6] Marquardt H., Hunkapiller M.W., Hood L.E., Twardzik D.R., De Larco J.E., Stephenson J.R., Todaro G.J. (1983). Transforming growth factors produced by retrovirus-transformed rodent fibroblasts and human melanoma cells: Amino acid sequence homology with epidermal growth factor. Proc. Natl. Acad. Sci. USA.

[7] Higashiyama S., Abraham J.A., Miller J., Fiddes J.C., Klagsbrun M. (1991). A heparin-binding growth factor secreted by macrophage-like cells that is related to EGF. Science.

[8] Ciardiello F., Kim N., Saeki T., Dono R., Persico M.G., Plowman G.D., Garrigues J., Radke S., Todaro G.J., Salomon D.S. (1991). Differential expression of epidermal growth factor-related proteins in human colorectal tumors. Proc. Natl. Acad. Sci. USA.

[9] Sasada R., Ono Y., Taniyama Y., Shing Y., Folkman J., Igarashi K. (1993). Cloning and expression of cDNA encoding human betacellulin, a new member of the EGF family. Biochem. Biophys. Res. Commun..

[10] Olayioye M.A., Neve R.M., Lane H.A., Hynes N.E. (2000). The ErbB signaling network: Receptor heterodimerization in development and cancer. EMBO J..

[11] Alroy I., Yarden Y. (1997). The ErbB signaling network in embryogenesis and oncogenesis: Signal diversification through combinatorial ligand-receptor interactions. FEBS Lett..

[12] Lewis T.S., Shapiro P.S., Ahn N.G. (1998). Signal transduction through MAP kinase cascades. Adv. Cancer Res..

[13] Hennessy B.T., Smith D.L., Ram P.T., Lu Y., Mills G.B. (2005). Exploiting the PI3K/AKT pathway for cancer drug discovery. Nat. Rev. Drug Discov..

[14] Silva C.M. (2004). Role of STATs as downstream signal transducers in Src family kinase-mediated tumorigenesis. Oncogene.

[15] Pensa S., Regis G., Boselli D., Novelli G., Poli V. (2009). STAT1 and STAT3 in Tumorigenesis: Two sides of the same coin?. Madame Curie Bioscience Database.

[16] Salomon D.S., Brandt R., Ciardiello F., Normanno N. (1995). Epidermal growth factor-related peptides and their receptors in human malignancies. Crit. Rev. Oncol. Hematol..

[17] Humblet Y. (2004). Cetuximab: An IgG(1) monoclonal antibody for the treatment of epidermal growth factor receptor-expressing tumours. Expert Opin. Pharmacother..

[18] Harding J., Burtness B. (2005). Cetuximab: An epidermal growth factor receptor chemeric human-murine monoclonal antibody. Drugs Today.

[19] Santiago A., Eicheler W., Bussink J., Rijken P., Yaromina A., Beuthien-Baumann B., van der Kogel A.J., Baumann M., Krause M. (2010). Effect of cetuximab and fractionated irradiation on tumour micro-environment. Radiother. Oncol..

[20] Naramura M., Gillies S.D., Mendelsohn J., Reisfeld R.A., Mueller B.M. (1993). Therapeutic potential of chimeric and murine anti-(epidermal growth factor receptor) antibodies in a metastasis model for human melanoma. Cancer Immunol. Immunother..

[21] Goldstein N.I., Prewett M., Zuklys K., Rockwell P., Mendelsohn J. (1995). Biological efficacy of a chimeric antibody to the epidermal growth factor receptor in a human tumor xenograft model. Clin. Cancer Res..

[22] De Bono J.S., Rowinsky E.K. (2002). The ErbB receptor family: A therapeutic target for cancer. Trends Mol. Med..

[23] Wu X., Rubin M., Fan Z., DeBlasio T., Soos T., Koff A., Mendelsohn J. (1996). Involvement of p27KIP1 in G1 arrest mediated by an anti-epidermal growth factor receptor monoclonal antibody. Oncogene.

[24] Peng D., Fan Z., Lu Y., De Blasio T., Scher H., Mendelsohn J. (1996). Anti-epidermal growth factor receptor monoclonal antibody 225 up-regulates p27KIP1 and induces G1 arrest in prostatic cancer cell line DU145. Cancer Res..

[25] Huang S.M., Bock J.M., Harari P.M. (1999). Epidermal growth factor receptor blockade with C225 modulates proliferation, apoptosis, and radiosensitivity in squamous cell carcinomas of the head and neck. Cancer Res..

[26] Baumann M., Krause M., Dikomey E., Dittmann K., Dörr W., Kasten-Pisula U., Rodemann H.P. (2007). EGFR-targeted anti-cancer drugs in radiotherapy: Preclinical evaluation of mechanisms. Radiother. Oncol..

[27] Vermorken J.B., Mesia R., Rivera F., Remenar E., Kawecki A., Rottey S., Erfan J., Zabolotnyy D., Kienzer H.R., Cupissol D. (2008). Platinum-based chemotherapy plus cetuximab in head and neck cancer. N. Engl. J. Med..

[28] Van Cutsem E., Köhne C.H., Hitre E., Zaluski J., Chang Chien C.R., Makhson A., D’Haens G., Pintér T., Lim R., Bodoky G. (2009). Cetuximab and chemotherapy as initial treatment for metastatic colorectal cancer. N. Engl. J. Med..

[29] Pan Q., Gorin M.A., Teknos T.N. (2009). Pharmacotherapy of head and neck squamous cell carcinoma. Expert. Opin. Pharmacother..

[30] Socinski M.A., Evans T., Gettinger S., Hensing T.A., Sequist L.V., Ireland B., Stinchcombe T.E. (2013). Treatment of stage IV non-small cell lung cancer: Diagnosis and management of lung cancer, 3rd ed; American College of Chest Physicians evidence-based clinical practice guidelines. Chest.

[31] Faloppi L., Andrikou K., Cascinu S. (2013). Cetuximab: Still an option in the treatment of pancreatic cancer?. Expert Opin. Biol. Ther..

[32] Bonner J.A., Harari P.M., Giralt J., Cohen R.B., Jones C.U., Sur R.K., Raben D., Baselga J., Spencer S.A., Zhu J. (2010). Radiotherapy plus cetuximab for locoregionally advanced head and neck cancer: 5-year survival data from a phase 3 randomised trial, and relation between cetuximab-induced rash and survival. Lancet Oncol..

[33] Bernier J., Schneider D. (2007). Cetuximab combined with radiotherapy: An alternative to chemoradiotherapy for patients with locally advanced squamous cell carcinomas of the head and neck?. Eur. J. Cancer.

[34] Caudell J.J., Sawrie S.M., Spencer S.A., Desmond R.A., Carroll W.R., Peters G.E., Nabell L.M., Meredith R.F., Bonner J.A. (2008). Locoregionally advanced head and neck cancer treated with primary radiotherapy: A comparison of the addition of cetuximab or chemotherapy and the impact of protocol treatment. Int. J. Radiat. Oncol. Biol. Phys..

[35] Agulnik M. (2012). New approaches to EGFR inhibition for locally advanced or metastatic squamous cell carcinoma of the head and neck (SCCHN). Med. Oncol..

[36] Robert F., Ezekiel M.P., Spencer S.A., Meredith R.F., Bonner J.A., Khazaeli M.B., Saleh M.N., Carey D., LoBuglio A.F., Wheeler R.H. (2001). Phase I study of anti-epidermal growth factor receptor antibody cetuximab in combination with radiation therapy in patients with advanced head and neck cancer. J. Clin. Oncol..

[37] Dattatreya S., Goswami C. (2011). Cetuximab plus radiotherapy in patients with unresectable locally advanced squamous cell carcinoma of head and neck region—A open labelled single arm phase II study. Indian J. Cancer.

[38] Ang K.K., Zhang Q.E., Rosenthal D.I., Nguyen-Tan P., Sherman E.J., Weber R.S., Galvin J.M., Schwartz D.L., El-Naggar A.K., Gillison M.L. (2011). A randomized phase III trial (RTOG 0522) of concurrent accelerated radiation plus cisplatin with or without cetuximab for stage III-IV head and neck squamous cell carcinomas (HNC). J. Clin. Oncol..

[39] Eriksen J.G., Maare C., Johansen J., Primdahl H., Evensen J., Kristensen C.A., Andersen L.J., Overgaard J. (2013). A randomized phase III study of primary curative (chemo)-radiotherapy and the egfr-inhibitor zalutumumab for squamous cell carcinoma of the head and neck (HNSCC). ESMO.

[40] Walsh L., Gillham C., Dunne M., Fraser I., Hollywood D., Armstrong J., Thirion P. (2011). Toxicity of cetuximab versus cisplatin concurrent with radiotherapy in locally advanced head and neck squamous cell cancer (LAHNSCC). Radiother. Oncol..

[41] Gurtner K., Deuse Y., Bütof R., Schaal K., Eicheler W., Oertel R., Grenman R., Thames H., Yaromina A., Baumann M. (2011). Diverse effects of combined radiotherapy and EGFR inhibition with antibodies or TK inhibitors on local tumour control and correlation with EGFR gene expression. Radiother. Oncol..

[42] Stegeman H., Kaanders J.H., van der Kogel A.J., Iida M., Wheeler D.L., Span P.N., Bussink J. (2013). Predictive value of hypoxia, proliferation and tyrosine kinase receptors for EGFR-inhibition and radiotherapy sensitivity in head and neck cancer models. Radiother. Oncol..

[43] Sok J.C., Coppelli F.M., Thomas S.M., Lango M.N., Xi S., Hunt J.L., Freilino M.L., Graner M.W., Wikstrand C.J., Bigner D.D. (2006). Mutant epidermal growth factor receptor (EGFRvIII) contributes to head and neck cancer growth and resistance to EGFR targeting. Clin. Cancer Res..

[44] Chen L.F., Cohen E.E., Grandis J.R. (2010). New strategies in head and neck cancer: Understanding resistance to epidermal growth factor receptor inhibitors. Clin. Cancer Res..

[45] Hubbard J.M., Alberts S.R. (2013). Alternate dosing of cetuximab for patients with metastatic colorectal cancer. Gastrointest. Cancer Res..

[46] Smilek P., Neuwirthova J., Jarkovsky J., Dusek L., Rottenberg J., Kostrica R., Srovnal J., Hajduch M., Drabek J., Klozar J. (2012). Epidermal growth factor receptor (EGFR) expression and mutations in the EGFR signaling pathway in correlation with anti-EGFR therapy in head and neck squamous cell carcinomas. Neoplasma.

[47] Bardelli A., Jänne P.A. (2012). The road to resistance: EGFR mutation and cetuximab. Nat. Med..

[48] Corcoran E.B., Hanson R.N. (2013). Imaging EGFR and HER2 by PET and SPECT: A Review. Med. Res. Rev..

[49] LNHB. http://www.nucleide.org/DDEP_WG/DDEPdata.htm.

[50] Lubberink M., Herzog H. (2011). Quantitative imaging of ^124^I and ^86^Y with PET. Eur. J. Nucl. Med. Mol. Imaging..

[51] FDA Data Specification. http://www.accessdata.fda.gov/drugsatfda_docs/label/2009/125084s168lbl.pdf.

[52] Milenic D.E., Brady E.D., Brechbiel M.W. (2004). Antibody-targeted radiation cancer therapy. Nat. Rev. Drug Discov..

[53] Srivastava S., Dadachova E. (2001). Recent advances in radionuclide therapy. Semin. Nucl. Med..

[54] Guo Y., Parry J.J., Laforest R., Rogers B.E., Anderson C.J. (2013). The role of p53 in combination radioimmunotherapy with ^64^Cu-DOTA-cetuximab and cisplatin in a mouse model of colorectal cancer. J. Nucl. Med..

[55] Szelecsenyi F., Blessing G., Qaim S.M. (1993). Excitation function of proton induced nuclear reactions on enriched ^61^Ni and ^64^Ni: Possibility of production of no-carrier-added ^61^Cu and ^64^Cu at a small cyclotron. Appl. Radiat. Isot..

[56] McCarthy D.W., Shefer R.E., Klinkowstein R.E., Bass L.A., Margeneau W.H., Cutler C.S., Anderson C.J., Welch M.J. (1997). Efficient production of high specific activity ^64^Cu using a biomedical cyclotron. Nucl. Med. Biol..

[57] Linder M.C., Hazegh-Azam M. (1996). Copper biochemistry and molecular biology. Am. J. Clin. Nutr..

[58] Anderson C.J., Ferdani R. (2009). Copper-64 radiopharmaceuticals for PET imaging of cancer: Advances in preclinical and clinical research. Cancer Biother. Radiopharm..

[59] Cai W., Chen K., He L., Cao Q., Koong A., Chen X. (2007). Quantitative PET of EGFR expression in xenograft-bearing mice using ^64^Cu-labeled cetuximab, a chimeric anti-EGFR monoclonal antibody. Eur. J. Nucl. Med. Mol. Imaging.

[60] Eiblmaier M., Meyer L.A., Watson M.A., Fracasso P.M., Pike L.J., Anderson C.J. (2008). Correlating EGFR expression with receptor-binding properties and internalization of ^64^Cu-DOTA-cetuximab in 5 cervical cancer cell lines. J. Nucl. Med..

[61] Ping Li W., Meyer L.A., Capretto D.A., Sherman C.D., Anderson C.J. (2008). Receptor-binding, biodistribution, and metabolism studies of ^64^Cu-DOTA-cetuximab, a PET-imaging agent for epidermal growth-factor receptor-positive tumors. Cancer Biother. Radiopharm..

[62] Niu G., Li Z., Xie J., Le Q.T., Chen X. (2009). PET of EGFR antibody distribution in head and neck squamous cell carcinoma models. J. Nucl. Med..

[63] Zhang Y., Hong H., Engle J.W., Bean J., Yang Y., Leigh B.R., Barnhart T.E., Cai W. (2011). Positron emission tomography imaging of CD105 expression with a ^64^Cu-labeled monoclonal antibody: NOTA is superior to DOTA. PLoS One.

[64] Velikyan I., Sundberg A.L., Lindhe O., Höglund A.U., Eriksson O., Werner E., Carlsson J., Bergström M., Långström B., Tolmachev V. (2005). Preparation and evaluation of ^68^Ga-DOTA-hEGF for visualization of EGFR expression in malignant tumors. J. Nucl. Med..

[65] Liu Z., Cui L., Liu X., Wang F. (2012). Noninvasive small-animal PET of trastuzumab-mediated EGFR down-regulation with ^68^Ga-Vec(Fab’)2. J. Nucl. Med..

[66] Strand J., Honarvar H., Perols A., Orlova A., Selvaraju R.K., Karlström A.E., Tolmachev V. (2013). Influence of macrocyclic chelators on the targeting properties of ^68^Ga-labeled synthetic affibody molecules: Comparison with ^111^In-labeled counterparts. PLoS One.

[67] Vosjan M.J., Perk L.R., Roovers R.C., Visser G.W., Stigter-van Walsum M., van Bergen E., Henegouwen P.M., van Dongen G.A. (2011). Facile labelling of an anti-epidermal growth factor receptor Nanobody with ^68^Ga via a novel bifunctional desferal chelate for immuno-PET. Eur. J. Nucl. Med. Mol. Imaging.

[68] Griffiths G.L., Chang C.H., McBride W.J., Rossi E.A., Sheerin A., Tejada G.R., Karacay H., Sharkey R.M., Horak I.D., Hansen H.J. (2004). Reagents and methods for PET using bispecific antibody pretargeting and 68Ga-radiolabeled bivalent hapten-peptide-chelate conjugates. J. Nucl. Med..

[69] Schuhmacher J., Klivényi G., Kaul S., Henze M., Matys R., Hauser H., Clorius J. (2001). Pretargeting of human mammary carcinoma xenografts with bispecific anti-MUC1/anti-Ga chelate antibodies and immunoscintigraphy with PET. Nucl. Med. Biol..

[70] Kuijpers W.H., Bos E.S., Kaspersen F.M., Veeneman G.H., van Boeckel C.A. (1993). Specific recognition of antibody-oligonucleotide conjugates by radiolabeled antisense nucleotides: A novel approach for two-step radioimmunotherapy of cancer. Bioconjug. Chem..

[71] Rusckowski M., Qu T., Chang F., Hnatowich D.J. (1997). Pretargeting using peptide nucleic acid. Cancer.

[72] Engle J.W., Hong H., Zhang Y., Valdovinos H.F., Myklejord D.V., Barnhart T.E., Theuer C.P., Nickles R.J., Cai W. (2012). Positron Emission Tomography Imaging of Tumor Angiogenesis with a ^66^Ga-Labeled Monoclonal Antibody. Mol. Pharm..

[73] Garmestani K., Milenic D.E., Plascjak P.S., Brechbiel M.W. (2002). A new and convenient method for purification of ^86^Y using a Sr(II) selective resin and comparison of biodistribution of ^86^Y and ^111^In labeled Herceptin. Nucl. Med. Biol..

[74] Perk L.R., Visser G.W., Vosjan M.J., Stigter-van Walsum M., Tijink B.M., Leemans C.R., van Dongen G.A. (2005). ^89^Zr as a PET surrogate radioisotope for scouting biodistribution of the therapeutic radiometals ^90^Y and ^177^Lu in tumor-bearing nude mice after coupling to the internalizing antibody cetuximab. J. Nucl. Med..

[75] Pentlow K.S., Finn R.D., Larson S.M., Erdi Y.E., Beattie B.J., Humm J.L. (2000). Quantitative Imaging of Yttrium-86 with PET. The Occurrence and Correction of Anomalous Apparent Activity in High Density Regions. Clin. Positron Imaging.

[76] Walrand S., Jamar F., Mathieu I., de camps J., Lonneux M., Sibomana M., Labar D., Michel C., Pauwels S. (2003). Quantitation in PET using isotopes emitting prompt single gammas: Application to yttrium-86. Eur. J. Nucl. Med. Mol. Imaging.

[77] Nayak T.K., Brechbiel M.W. (2009). Radioimmunoimaging with longer-lived positron-emitting radionuclides: Potentials and challenges. Bioconjug Chem..

[78] Lövqvist A., Humm J.L., Sheikh A., Finn R.D., Koziorowski J., Ruan S., Pentlow K.S., Jungbluth A., Welt S., Lee F.T. (2001). PET imaging of ^86^Y-labe.led anti-Lewis Y monoclonal antibodies in a nude mouse model: Comparison between ^86^Y and (111)In radiolabels. J. Nucl. Med..

[79] Palm S., Enmon R.M., Matei C., Kolbert K.S., Xu S., Zanzonico P.B., Finn R.L., Koutcher J.A., Larson S.M., Sgouros G. (2003). Pharmacokinetics and Biodistribution of ^86^Y-Trastuzumab for ^90^Y dosimetry in an ovarian carcinoma model: Correlative MicroPET and MRI. J. Nucl. Med..

[80] Schneider D.W., Heitner T., Alicke B., Light D.R., McLean K., Satozawa N., Parry G., Yoo J., Lewis J.S., Parry R. (2009). *In vivo* biodistribution, PET imaging, and tumor accumulation of ^86^Y- and ^111^In-antimindin/RG-1, engineered antibody fragments in LNCaP tumor-bearing nude mice. J. Nucl. Med..

[81] Nayak T.K., Garmestani K., Baidoo K.E., Milenic D.E., Brechbiel M.W. (2010). Preparation, biological evaluation, and pharmacokinetics of the human anti-HER1 monoclonal antibody panitumumab labeled with ^86^Y for quantitative PET of carcinoma. J. Nucl. Med..

[82] Wong K.J., Baidoo K.E., Nayak T.K., Garmestani K., Brechbiel M.W., Milenic D.E. (2011). *In Vitro* and *In Vivo* Pre-Clinical Analysis of a F(ab')(2) Fragment of Panitumumab for Molecular Imaging and Therapy of HER1 Positive Cancers. EJNMMI Res..

[83] Nayak T.K., Regino C.A., Wong K.J., Milenic D.E., Garmestani K., Baidoo K.E., Szajek L.P., Brechbiel M.W. (2010). PET imaging of HER1-expressing xenografts in mice with ^86^Y-CHX-A′′-DTPA-cetuximab. Eur. J. Nucl. Med. Mol. Imaging.

[84] Nayak T.K., Garmestani K., Milenic D.E., Baidoo K.E., Brechbiel M.W. (2011). HER1-targeted ^86^Y-panitumumab possesses superior targeting characteristics than ^86^Y-cetuximab for PET imaging of human malignant mesothelioma tumors xenografts. PLoS One.

[85] Milenic D.E., Wong K.J., Baidoo K.E., Ray G.L., Garmestani K., Williams M., Brechbiel M.W. (2008). Cetuximab: Preclinical evaluation of a monoclonal antibody targeting EGFR for radioimmunodiagnostic and radioimmunotherapeutic applications. Cancer Biother Radiopharm..

[86] Verel I., Visser G.W., Boellaard R., Stigter-van Walsum M., Snow G.B., van Dongen G.A. (2003). ^89^Zr immuno-PET: Comprehensive procedures for the production of ^89^Zr-labeled monoclonal antibodies. J. Nucl. Med..

[87] FDA-Specification. http://www.accessdata.fda.gov/drugsatfda_docs/label/2012/125084s225lbl.pdf.

[88] Börjesson P.K., Jauw Y.W., de Bree R., Roos J.C., Castelijns J.A., Leemans C.R., van Dongen G.A., Boellaard R. (2009). Radiation dosimetry of ^89^Zr-labeled chimeric monoclonal antibody U36 as used for immuno-PET in head and neck cancer patients. J. Nucl. Med..

[89] Perk L.R., Visser O.J., Stigter-van Walsum M., Vosjan M.J., Visser G.W., Zijlstra J.M., Huijgens P.C., van Dongen G.A. (2006). Preparation and evaluation of ^89^Zr-Zevalin for monitoring of ^90^Y-Zevalin biodistribution with positron emission tomography. Eur. J. Nucl. Med. Mol. Imaging.

[90] Van Nerom C.G., Bormans G.M., de Roo M.J., Verbruggen A.M. (1993). First experience in healthy volunteers with technetium-99m L,L-ethylenedicysteine, a new renal imaging agent. Eur. J. Nucl. Med..

[91] Schechter N.R., Yang D.J., Azhdarinia A., Kohanim S., Wendt R., Oh C.S., Hu M., Yu D.F., Bryant J., Ang K.K. (2003). Assessment of epidermal growth factor receptor with ^99m^Tc-ethylenedicysteine-C225 monoclonal antibody. Anticancer Drugs.

[92] Schechter N.R., Wendt R.E., Yang D.J., Azhdarinia A., Erwin W.D., Stachowiak A.M., Broemeling L.D., Kim E.E., Cox J.D., Podoloff D.A. (2004). Radiation dosimetry of ^99m^Tc-labeled C225 in patients with squamous cell carcinoma of the head and neck. J. Nucl. Med..

[93] Kaur S., Venktaraman G., Jain M., Senapati S., Garg P.K., Batra S.K. (2012). Recent trends in antibody-based oncologic imaging. Cancer Lett..

[94] Capello A., Krenning E.P., Breeman W.A., Bernard B.F., de Jong M. (2003). Peptide receptor radionuclide therapy *in vitro* using [^111^In-DTPA0]octreotide. J. Nucl. Med..

[95] Huhtala T., Laakkonen P., Sallinen H., Ylä-Herttuala S., Närvänen A. (2010). *In vivo* SPECT/CT imaging of human orthotopic ovarian carcinoma xenografts with 111In-labeled monoclonal antibodies. Nucl. Med. Biol..

[96] Price E.W., Zeglis B.M., Cawthray J.F., Ramogida C.F., Ramos N., Lewis J.S., Adam M.J., Orvig C. (2013). H(4)octapa-trastuzumab: Versatile acyclic chelate system for ^111^In and ^177^Lu imaging and therapy. J. Am. Chem. Soc..

[97] Yoshida H., Mochizuki M., Kainouchi M., Ishida T., Sakata K., Yokoyama S., Hoshino T., Takezawa M., Matsumoto Y., Miyamoto T. (1991). Clinical application of indium-111 antimyosin antibody and thallium-201 dual nuclide single photon emission computed tomography in acute myocardial infarction. Ann. Nucl. Med..

[98] Divgi C.R., Welt S., Kris M., Real F.X., Yeh S.D., Gralla R., Merchant B., Schweighart S., Unger M., Larson S.M. (1991). Phase I and imaging trial of indium-111 labeled anti-epidermal growth factor receptor monoclonal antibody 225 in patients with squamous cell lung carcinoma. J. Natl. Cancer Inst..

[99] Dillman L.T., von der Lage F.C. (1975). NM/MIRD Pamphlet No. 10: Radionuclide Decay Schemes and Nuclear Parameters for Use in Radiation-Dose Estimation. New York. Soc. Nucl. Med..

[100] Bading J.R., Hörling M., Williams L.E., Colcher D., Raubitschek A., Strand S.E. (2008). Quantitative serial imaging of an 124I anti-CEA monoclonal antibody in tumor-bearing mice. Cancer Biother. Radiopharm..

[101] Yao M., Faulhaber P.F. (2012). PET imaging of the head and neck. PET Clinics.

[102] Lee F.T., Hall C., Rigopoulos A., Zweit J., Pathmaraj K., O’Keefe G.J., Smyth F.E., Welt S., Old L.J., Scott A.M. (2001). Immuno-PET of human colon xenograft- bearing BALB/c nude mice using ^124^I-CDR-grafted humanized A33 monoclonal antibody. J. Nucl. Med..

[103] Fortin M.A., Salnikov A.V., Nestor M., Heldin N.E., Rubin K., Lundqvist H. (2007). Immuno-PET of undifferentiated thyroid carcinoma with radioiodine-labelled antibody cMAb U36: Application to antibody tumour uptake studies. Eur. J. Nucl. Med. Mol. Imaging.

[104] Lee F.T., O’Keefe G.J., Gan H.K., Mountain A.J., Jones G.R., Saunder T.H., Sagona J., Rigopoulos A., Smyth F.E., Johns T.G. (2010). Immuno-PET quantitation of de2-7 epidermal growth factor receptor expression in glioma using ^124^I-IMP-R4-labeled antibody ch806. J. Nucl. Med..

[105] Tijink B.M., Neri D., Leemans C.R., Budde M., Dinkelborg L.M., Stigter-van Walsum M., Zardi L., van Dongen G.A. (2006). Radioimmunotherapy of head and neck cancer xenografts using ^131^I-labeled antibody L19-SIP for selective targeting of tumor vasculature. J. Nucl. Med..

[106] Nestor M., Ekberg T., Dring J., van Dongen G.A., Wester K., Tolmachev V., Anniko M. (2007). Quantification of CD44v6 and EGFR expression in head and neck squamous cell carcinomas using a single-dose radioimmunoassay. Tumour Biol..

[107] Nordberg E., Friedman M., Göstring L., Adams G.P., Brismar H., Nilsson F.Y., Ståhl S., Glimelius B., Carlsson J. (2007). Cellular studies of binding, internalization and retention of a radiolabeled EGFR-binding affibody molecule. Nucl. Med. Biol..

[108] Hoeben B.A., Molkenboer-Kuenen J.D., Oyen W.J., Peeters W.J., Kaanders J.H., Bussink J., Boerman O.C. (2011). Radiolabeled cetuximab: Dose optimization for epidermal growth factor receptor imaging in a head-and-neck squamous cell carcinoma model. Int. J. Cancer.

[109] Tijink B.M., Laeremans T., Budde M., Stigter-van Walsum M., Dreier T., de Haard H.J., Leemans C.R., van Dongen G.A. (2008). Improved tumor targeting of anti-epidermal growth factor receptor Nanobodies through albumin binding: Taking advantage of modular Nanobody technology. Mol. Cancer Ther..

[110] Börjesson P.K., Postema E.J., de Bree R., Roos J.C., Leemans C.R., Kairemo K.J., van Dongen G.A. (2004). Radioimmunodetection and radioimmunotherapy of head and neck cancer. Oral. Oncol..

[111] Jowsey J., Rowland R.E., Marshall J.H. (1958). The deposition of the rare earths in bone. Radiat. Res..

[112] Minarik D., Ljungberg M., Segars P., Gleisner K.S. (2009). Evaluation of quantitative planar ^90^Y bremsstrahlung whole-body imaging. Phys. Med. Biol..

[113] Elschot M., Vermolen B.J., Lam M.G., de Keizer B., van den Bosch M.A., de Jong H.W. (2013). Quantitative comparison of PET and Bremsstrahlung SPECT for imaging the *in vivo* yttrium-90 microsphere distribution after liver radioembolization. PLoS One.

[114] Goodwin D.A., Meares C.F., Watanabe N., McTigue M., Chaovapong W., Ransone C.M., Renn O., Greiner D.P., Kukis D.L., Kronenberger S.I. (1994). Pharmacokinetics of pretargeted monoclonal antibody 2D12.5 and ^88^Y-Janus-2-(p-nitrobenzyl)-1,4,7,10-tetraazacyclododecanetetraacetic acid (DOTA) in BALB/c mice with KHJJ mouse adenocarcinoma: A model for ^90^Y radioimmunotherapy. Cancer Res..

[115] Postema E.J., Frielink C., Oyen W.J., Raemaekers J.M., Goldenberg D.M., Corstens F.H., Boerman O.C. (2003). Biodistribution of ^131^I-, ^186^Re-, ^177^Lu-, and ^88^Y-labeled hLL2 (Epratuzumab) in nude mice with CD22-positive lymphoma. Cancer Biother. Radiopharm..

[116] Walrand S., Flux G.D., Konijnenberg M.W., Valkema R., Krenning E.P., Lhommel R., Pauwels S., Jamar F. (2011). Dosimetry of yttrium-labelled radiopharmaceuticals for internal therapy: ^86^Y or ^90^Y imaging?. Eur. J. Nucl. Med. Mol. Imaging.

[117] Niu G., Sun X., Cao Q., Courter D., Koong A., Le Q.T., Gambhir S.S., Chen X. (2010). Cetuximab-based immunotherapy and radioimmunotherapy of head and neck squamous cell carcinoma. Clin. Cancer Res..

[118] Koi L., Bergmann R., Brüchner K., Pietzsch H.J., Krause M., Steinbach J., Zips D., Baumann M. (2014). Theragnostic radiolabeled EGFR-antibody improves local tumor control after external radiotherapy. Radiother. Oncol..

[119] Saki M., Toulany M., Sihver W., Zenker M., Heldt J.M., Mosch B., Pietzsch H.J., Baumann M., Steinbach J., Rodemann H.P. (2012). Cellular and molecular properties of ^90^Y-labeled cetuximab in combination with radiotherapy on human tumor cells *in vitro*. Strahlenther. Onkol..

[120] Verburg F.A., Luster M., Lassmann M., Reiners C. (2011). ^131^I therapy in patients with benign thyroid disease does not conclusively lead to a higher risk of subsequent malignancies. Nuklearmedizin.

[121] Grünwald F., Ezziddin S. (2010). ^131^I-metaiodobenzylguanidine therapy of neuroblastoma and other neuroendocrine tumors. Semin. Nuc. Med..

[122] Sisson J.C., Carey J.E. (2001). Thyroid carcinoma with high levels of function: Treatment with ^131^I. J. Nucl. Med..

[123] Xue Y.L., Qiu Z.L., Song H.J., Luo Q.Y. (2013). Value of ^131^I SPECT/CT for the evaluation of differentiated thyroid cancer: A systematic review of the literature. Eur. J. Nucl. Med. Mol. Imaging.

[124] Rades D., Wolff C., Nadrowitz R., Breunig C., Schild S.E., Baehre M., Meller B. (2009). Radioactive EGFR antibody cetuximab in multimodal cancer treatment: Stability and synergistic effects with radiotherapy. Int. J. Radiat. Oncol. Biol. Phys..

[125] Schlom J., Siler K., Milenic D.E., Eggensperger D., Colcher D., Miller L.S., Houchens D., Cheng R., Kaplan D., Goeckeler W. (1991). Monoclonal antibody-based therapy of a human tumor xenograft with a ^177^lutetium-labeled im munoconjugate. Cancer Res..

[126] Mulligan T., Carrasquillo J.A., Chung Y., Milenic D.E., Schlom J., Feuerstein I., Paik C., Perentesis P., Reynolds J., Curt G. (1995). Phase I study of intravenous Lu-labeled CC49 murine monoclonal antibody in patients with advanced adenocarcinoma. J. Clin. Cancer Res..

[127] Stein R., Govindan S.V., Chen S., Reed L., Richel H., Griffiths G.L., Hansen H.J., Goldenberg D.M. (2001). Radioimmunotherapy of a human lung cancer xenograft with monoclonal antibody RS7: Evaluation of ^177^Lu and comparison of its efficacy with that of ^90^Y and residualizing ^131^I. J. Nucl. Med..

[128] Lee S.Y., Hong Y.D., Kim H.S., Choi S.J. (2013). Synthesis and application of a novel cysteine-based DTPA-NCS for targeted radioimmunotherapy. Nucl. Med. Biol..

[129] Jurcic J.G., Larson S.M., Sgouros G., McDevitt M.R., Finn R.D., Divgi C.R., Ballangrud A.M., Hamacher K.A., Ma D., Humm J.L. (2002). Targeted alpha particle immunotherapy for myeloid leukemia. Blood.

[130] Song H., Shahverdi K., Huso D.L., Esaias C., Fox J., Liedy A., Zhang Z., Reilly R.T., Apostolidis C., Morgenstern A. (2008). ^213^Bi (alpha-emitter)-antibody targeting of breast cancer metastases in the neu-N transgenic mouse model. Cancer Res..

[131] Ma D., McDevitt M.R., Finn R.D., Scheinberg D.A. (2001). Breakthrough of ^225^Ac and its radionuclide daughters from an ^225^Ac/^213^Bi generator: Development of new methods, quantitative characterization, and implications for clinical use. Appl. Radiat. Isot..

[132] Rosenblat T.L., McDevitt M.R., Mulford D.A., Pandit-Taskar N., Divgi C.R., Panageas K.S., Heaney M.L., Chanel S., Morgenstern A., Sgouros G. (2010). Sequential cytarabine and alpha-particle immunotherapy with bismuth-213-lintuzumab (HuM195) for acute myeloid leukemia. Clin. Cancer Res..

[133] Andersson H., Cederkrantz E., Bäck T., Divgi C., Elgqvist J., Himmelman J., Horvath G., Jacobsson L., Jensen H., Lindegren S. (2009). Intraperitoneal alpha-particle radioimmunotherapy of ovarian cancer patients: Pharmacokinetics and dosimetry of ^211^At-MX35 F(ab')2—A phase I study. J. Nucl. Med..

[134] Allen B.J., Singla A.A., Rizvi S.M., Graham P., Bruchertseifer F., Apostolidis C., Morgenstern A. (2011). Analysis of patient survival in a Phase I trial of systemic targeted α-therapy for metastatic melanoma. Immunotherapy.

[135] Song H., Hedayati M., Hobbs R.F., Shao C., Bruchertseifer F., Morgenstern A., Deweese T.L., Sgouros G. (2013). Targeting aberrant DNA double strand break repair in triple negative breast cancer with alpha particle emitter radiolabeled anti-EGFR antibody. Mol. Cancer Ther..

[136] Brechbiel M.W. (2008). Bifunctional chelates for metal nuclides. Q. J. Nucl. Med. Mol. Imaging.

[137] Pritchard J.H., Ackerman M., Tubis M., Blahd W.H. (1976). Indium-111-labeled antibody heavy metal chelate conjugates: A potential alternative to radioiodination. Proc. Soc. Exp. Biol. Med..

[138] Ward M.C., Roberts K.R., Babich J.W., Bukhari M.A., Coghlan G., Westwood J.H., McCready V.R., Ott R.J. (1986). An antibody-desferrioxamine conjugate labelled with ^67^Ga. Int. J. Rad. Appl. Instrum. B.

[139] Perk L.R., Vosjan M.J., Visser G.W., Budde M., Jurek P., Kiefer G.E., van Dongen G.A. (2010). p-Isothiocyanatobenzyl-desferrioxamine: A new bifunctional chelate for facile radiolabeling of monoclonal antibodies with zirconium-89 for immuno-PET imaging. Eur. J. Nucl. Med. Mol. Imaging.

[140] Chang A.J., de Silva R.A., Lapi S.E. (2013). Development and characterization of ^89^Zr-labeled panitumumab for immuno-positron emission tomographic imaging of the epidermal growth factor receptor. Mol. Imaging.

[141] Aerts H.J., Dubois L., Perk L., Vermaelen P., van Dongen G.A., Wouters B.G., Lambin P. (2009). Disparity between *in vivo* EGFR expression and ^89^Zr-labeled cetuximab uptake assessed with PET. J. Nucl. Med..

[142] Karmani L., Labar D., Valembois V., Bouchat V., Nagaswaran P.G., Bol A., Gillart J., Levêque P., Bouzin C., Bonifazi D. (2013). Antibody-functionalized nanoparticles for imaging cancer: Influence of conjugation to gold nanoparticles on the biodistribution of ^89^Zr-labeled cetuximab in mice. Contrast Media Mol. Imaging.

[143] McMurry T.J., Pippin C.G., Wu C., Deal K.A., Brechbiel M.W., Mirzadeh S., Gansow O.A. (1998). Physical parameters and biological stability of yttrium(III) diethylenetriaminepentaacetic acid derivative conjugates. J. Med. Chem..

[144] Kobayashi H., Wu C., Yoo T.M., Sun B.F., Drumm D., Pastan I., Paik C.H., Gansow O.A., Carrasquillo J.A., Brechbiel M.W. (1998). Evaluation of the *in vivo* biodistribution of yttrium-labeled isomers of CHX-DTPA-conjugated monoclonal antibodies. J. Nucl. Med..

[145] Lee F.T., Mountain A.J., Kelly M.P., Hall C., Rigopoulos A., Johns T.G., Smyth F.E., Brechbiel M.W., Nice E.C., Burgess A.W. (2005). Enhanced efficacy of radioimmunotherapy with ^90^Y-CHX-A′′-DTPA-hu3S193 by inhibition of epidermal growth factor receptor (EGFR) signaling with EGFR tyrosine kinase inhibitor AG1478. Clin. Cancer Res..

[146] Fani M., Bouziotis P., Harris A.L., Psimadas D., Gourni E., Loudos G., Varvarigou A.D., Maecke H.R. (2007). ^177^Lu-labeled-VG76e monoclonal antibody in tumor angiogenesis: A comparative study using DOTA and DTPA chelating systems. Radiochim. Acta.

[147] Ray G.L., Baidoo K.E., Wong K.J., Williams M., Garmestani K., Brechbiel M.W., Milenic D.E. (2009). Preclinical evaluation of a monoclonal antibody targeting the epidermal growth factor receptor as a radioimmunodiagnostic and radioimmunotherapeutic agent. Br. J. Pharmacol..

[148] Boswell C.A., Sun X., Niu W., Weisman G.R., Wong E.H., Rheingold A.L., Anderson C.J. (2004). Comparative *in vivo* stability of copper-64-labeled cross-bridged and conventional tetraazamacrocyclic complexes. J. Med. Chem..

[149] Tolmachev V., Wållberg H., Andersson K., Wennborg A., Lundqvist H., Orlova A. (2009). The influence of Bz-DOTA and CHX-A′′-DTPA on the biodistribution of ABD-fused anti-HER2 Affibody molecules: implications for ^114m^In-mediated targeting therapy. Eur. J. Nucl. Med. Mol. Imaging..

[150] Milenic D.E., Garmestani K., Chappell L.L., Dadachova E., Yordanov A., Ma D., Schlom J., Brechbiel M.W. (2002). *In vivo* comparison of macrocyclic and acyclic ligands for radiolabeling of monoclonal antibodies with ^177^Lu for radioimmunotherapeutic applications. Nucl. Med. Biol..

[151] Niu G., Cai W., Chen K., Chen X. (2008). Non-invasive PET imaging of EGFR degradation induced by a heat shock protein 90 inhibitor. Mol. Imaging Biol..

[152] Delgado R., Sun Y., Motekaitis R.J., Martell A.E. (1993). Stabilities of divalent and trivalent metal ion complexes of macrocyclic triazatriacetic acids. Inorg. Chem..

[153] Sprague J.E., Peng Y., Sun X., Weisman G.R., Wong E.H., Achilefu S., Anderson C.J. (2004). Preparation and biological evaluation of copper-64-labeled Tyr3-Octreotate using a cross-bridged macrocyclic cheator. Clin. Cancer Res..

[154] Ingargiola M., Dittfeld C., Runge R., Zenker M., Heldt J.M., Steinbach J., Cordes N., Baumann M., Kotzerke J., Kunz-Schughart L.A. (2012). Flow cytometric cell-based assay to preselect antibody constructs for radionuclide conjugation. Cytometry A.

[155] Zhang Y., Hong H., Engle J.W., Yang Y., Theuer C.P., Barnhart T.E., Cai W. (2012). Positron Emission Tomography and Optical Imaging of Tumor CD105 Expression with a Dual-Labeled Monoclonal Antibody. Mol. Pharm..

[156] Cohen R., Stammes M.A., de Roos I.H., Stigter-van Walsum M., Visser G.W., van Dongen G.A. (2011). Inert coupling of IRDye800CW to monoclonal antibodies for clinical optical imaging of tumor targets. EJNMMI Res..

[157] Vakili A., Jalilian A.R., Yavari K., Shirvani-Arani S., Khanchi A., Bahrami-Samani A., Salimi B., Khorrami-Moghadam A. (2013). Preparation and quality control and biodistribution studies of [^90^Y]-DOTA-cetuximab for radioimmunotherapy. J. Radioanal. Nucl. Chem..

[158] Wen X., Wu Q.P., Ke S., Ellis L., Charnsangavej C., Delpassand A.S., Wallace S., Li C. (2001). Conjugation with ^111^In-DTPA-poly(ethylene glycol) improves imaging of anti-EGF receptor antibody C225. J. Nucl. Med..

[159] Van Dijk L.K., Hoeben B.A., Stegeman H., Kaanders J.H., Franssen G.M., Boerman O.C., Bussink J. (2013). ^111^In-cetuximab-F(ab')2 SPECT imaging for quantification of accessible epidermal growth factor receptors (EGFR) in HNSCC xenografts. Radiother. Oncol..

[160] Van Dongen G.A., Visser G.W., Lub-de Hooge M.N., de Vries E.G., Perk L.R. (2007). Immuno-PET: A navigator in monoclonal antibody development and applications. Oncologist.

[161] Walrand S., Barone R., Pauwels S., Jamar F. (2011). Experimental facts supporting a red marrow uptake due to radiometal transchelation in ^90^Y-DOTATOC therapy and relationship to the decrease of platelet counts. Eur. J. Nucl. Med. Mol. Imaging.

[162] Vakili A., Jalilian A.R., Moghadam A.K., Ghazi-Zahedi M., Salimi B. (2012). Evaluation and comparison of human absorbed dose of ^90^Y-DOTA-Cetuximab in various age groups based on distribution data in rats. J. Med. Phys..

[163] Pilaro A.M. (2003). Pharmacology/toxicology review and evaluation. Erbitux. Accessdata FDA Application number STN/BLA 125084. Cent. Drug Eval. Res..

[164] Matsumura Y., Maeda H. (1986). A new concept for macromolecular therapeutics in cancer chemotherapy: Mechanism of tumoritropic accumulation of proteins and the antitumor agent smancs. Cancer Res..

[165] Maeda H., Nakamura H., Fang J. (2013). The EPR effect for macromolecular drug delivery to solid tumors: Improvement of tumor uptake, lowering of systemic toxicity, and distinct tumor imaging *in vivo*. Adv. Drug. Deliv. Rev..

[166] Ogawa M., Regino C.A., Choyke P.L., Kobayashi H. (2009). *In vivo* target-specific activatable near-infrared optical labeling of humanized monoclonal antibodies. Mol. Cancer Ther..

[167] Maeda H., Bharate G.Y., Daruwalla J. (2009). Polymeric drugs for efficient tumor-targeted drug delivery based on EPR-effect. Eur. J. Pharm. Biopharm..

[168] Perera R.M., Zoncu R., Johns T.G., Pypaert M., Lee F.T., Mellman I., Old L.J., Toomre D.K., Scott A.M. (2007). Internalization, intracellular trafficking, and biodistribution of monoclonal antibody 806: A novel anti-epidermal growth factor receptor antibody. Neoplasia.

[169] Oude Munnink T.H., Tamas K.R., Lub-de Hooge M.N., Vedelaar S.R., Timmer-Bosscha H., Walenkamp A.M., Weidner K.M., Herting F., Tessier J., de Vries E.G. (2013). Placental growth factor (PlGF)-specific uptake in tumor microenvironment of ^89^Zr-labeled PlGF antibody RO5323441. J. Nucl. Med..

[170] Danhier F., Feron O., Préat V. (2010). To exploit the tumor microenvironment: Passive and active tumor targeting of nanocarriers for anti-cancer drug delivery. J. Control Release.

[171] Dittmann K., Mayer C., Rodemann H.P. (2005). Inhibition of radiation-induced EGFR nuclear import by C225 (Cetuximab) suppresses DNA-PK activity. Radiother. Oncol..

[172] Karar J., Maity A. (2009). Modulating the tumor microenvironment to increase radiation responsiveness. Cancer Biol. Ther..

[173] Saker J., Kriegs M., Zenker M., Heldt J.M., Eke I., Pietzsch H.J., Grénman R., Cordes N., Petersen C., Baumann M. (2013). Inactivation of HNSCC cells by ^90^Y-labeled cetuximab strictly depends on the number of induced DNA double-strand breaks. J. Nucl. Med..

[174] Krause M., Ostermann G., Petersen C., Yaromina A., Hessel F., Harstrick A., van der Kogel A.J., Thames H.D., Baumann M. (2005). Decreased repopulation as well as increased reoxygenation contribute to the improvement in local control after targeting of the EGFR by C225 during fractionated irradiation. Radiother. Oncol..

[175] Eiblmaier M., Meyer L.A., Anderson C.J. (2008). The role of p53 in the trafficking of copper-64 to tumor cell nuclei. Cancer Biol. Ther..

[176] Rades D., Nadrowitz R., Buchmann I., Hunold P., Noack F., Schild S.E., Meller B. (2010). Radiolabeled cetuximab plus whole-brain irradiation (WBI) for the treatment of brain metastases from non-small cell lung cancer (NSCLC). Strahlenther. Onkol..

